# Player involvement as a result of difficulty: An introductory study to test the suitability of the motivational intensity approach to video game research

**DOI:** 10.1371/journal.pone.0282966

**Published:** 2023-03-10

**Authors:** Paweł Strojny, Agnieszka Strojny, Krzysztof Rębilas

**Affiliations:** 1 Faculty of Management and Social Communication Institute of Applied Psychology, Jagiellonian University in Cracow, Kraków, Poland; 2 Doctoral School in the Social Sciences, Jagiellonian University in Cracow, Kraków, Poland; PLOS ONE, UNITED KINGDOM

## Abstract

Motivational Intensity Theory could serve as a useful framework in the process of analyzing and optimizing a user’s involvement in computer games. However, it has not yet been used in this way. Its main advantage is that it makes clear predictions regarding the relations between difficulty level, motivation and commitment. The current study aimed to test whether the postulates of this theory may be useful in the process of game development. Forty-two participants took part in a fully controlled within-subjects experiment utilizing a commonly available game (Icy Tower) that has several levels of difficulty. Participants played on four increasing levels of difficulty and their task was to play as best they could, with the aim of reaching the hundredth platform. As a result, we demonstrated that involvement level increases as the difficulty level increases when a task is feasible, but it drops rapidly when a task is so difficult that it cannot be completed. This is the very first evidence that Motivational Intensity Theory may be useful in game research and design. The following study also supports concerns regarding the usefulness of self-report data in the game design process.

## Introduction

‘Why is a given gamer playing a given game and not another?’ This question prompts numerous researchers and practitioners to try to identify the personality- and game-related factors that make certain titles a good fit for certain users. Researchers working in this field try to understand players’ behavior using numerous constructs and theories, among the most popular of which are a game’s ability to satisfy the needs of a player [1–4], the process of learning [5], or flow theory [6]. The objective of this approach is to discover the conditions that favor the internal [7] (or endogenous [8]) motivation that drives behavior. Researchers create and improve classifications of the specific motives that determine a player’s willingness to play certain types of games [9, 10]. Connecting specific motives with gameplay characteristics is the basis of predicting involvement in a game [10] or a game’s effects on an individual’s well-being [11]. We understand involvement here as willingness to invest resources into an activity, as considering getting involved in something always requires investing time, energy or material resources in order to achieve expected results. These and numerous other studies are part of a trend based on uses and gratifications theory [12], which stipulates that consumers of media (including gamers) are active, goal-oriented, conscious users who, guided by their own judgement, choose from competing media those which give them the greatest gratification [13]. This is done through the development of various motivations for their media consumption [14]. As a consequence, most of the related research assumes that these constructs remain constant. This assumption drives researchers to seek relationships between relatively constant game and user characteristics, thereby allowing us to draw useful but incomplete conclusions. Establishing a list of motives for gaming or a list of the needs that a game fulfills is a static approach, it is not due to the nature of motives but due to the misunderstanding of the motivation, relatively frequent in the domain of gaming research. Although motives as such have dynamics, for example, they can be activated or satisfied, the way they are measured (a questionnaire containing a list of motives for which a given person plays) means that the picture that emerges from such research may simplify the real situation. A player may stop playing both because their motives were temporarily satisfied, but also because they felt the particular game has stopped satisfying them. The first to notice this was Shand, who stated that “Each study is a snapshot, a still image of user motivations at one moment in time. But is that how motivations for media use exist–as constant, unchanging variables?” [15]. The vast majority of gamers do not play all the time [16]. The right question to be answered in order to understand the dynamic determinants of engaging in gaming should be ‘Why is a given gamer playing a given game at a certain moment?’. Thus, the research problem we decided to address concerns the factors that may influence momentary involvement in a game, regardless of the configuration of the relatively static variables that determine game preferences. Identifying this group of factors may be crucial for objectives such as counteracting gaming disorder or increasing involvement in professional games and simulators. The goal of the current paper is to show that this approach needs to be supplemented with an obvious but mostly overlooked element: the factors that determine momentary involvement in gaming. We present a theoretical framework that allows this to be explored and we offer the first empirical evidence for the proposed approach.

### The research on gamers’ motivations and its limitations

Research on motivation toward computer gaming became separate from other media research when online multiplayer games became popular. In around 2005, researchers considered the possible effects of gaming motivations on in-game and out-of-game behavior. For example, Vorderer, et al. [17] examined the relationship between individual motivations and the preference for violent games. Other authors searched for relationships between gaming motivations and other variables, such as gender, and other demographic differences [6, 14], gaming habits [18], preferences for game genres [19], well-being [20], gaming disorder [21] and many others. What all of these studies had in common is the assumption that the reasons people play games and the games they choose may be related not only to their gaming preferences but also to other relatively constant variables. Studies in this paradigm have resulted in numerous taxonomies of gamers’ motivations; these have been reviewed, for example, in Herodotou et al. [22], while some of the most prominent classifications are Yee’s [9] and Demetrovics’ [23] models. However, when observing the behavior of gamers, it cannot be concluded that all we need to explain them is a static taxonomy [15].

The first researchers who admitted the possibility of taking dynamic factors into account were Reinhard and Dervin [14], who distinguished three different gaming situations (playing favorite, disliked and desirable games) and identified the accompanying motivations. Also, Schultheiss [24] found that at least some motivations change over time. However, it was only Shand [15] who explicitly indicated such a necessity and conducted research aimed at identifying the motivations that accompany contact with games at three stages (starting, continuing and quitting a game). Shand’s work was continued by Carradini and Hommadova [25]. This lack of interest in the subject of dynamic changes in gaming motivation may be surprising: after all, both game developers and institutions that offer prevention methods and therapy for gaming disorder could benefit greatly from a more precise understanding of the motives behind gaming. As Carradini and Hommadova [25] suggest, the reason for such a state of affairs may lay both in the dominance of methods that promote a cross-sectional approach, as well as in theoretical assumptions regarding the determinants of gaming involvement.

Undoubtedly, gamers’ involvement fluctuates within the periods distinguished in earlier studies. We believe that the examples of research on gamers’ involvement cited above have added great value to our understanding of this important area. However, they also have a serious limitation—they focus on relatively stable variables, and even if they allow a certain change in involvement over time, the methods used and theoretical foundations do not allow them to move to the dynamics in minute or even second resolution. Thus, another step is needed to understand the temporal dynamics of gaming involvement. We would like to draw attention to the necessity and possibility of studying dynamic changes in gamers’ involvement with a resolution of minutes. This is a necessary condition to understand the general reasons why people decide to play video games and to recognize the fact that gaming motivators alternate between initial fascination and weariness with a game. To adopt such a perspective, it is necessary to change the theoretical foundations and, to some extent, the methodology. This paper proposes the use of Motivational Intensity Theory in the domain of gamers’ involvement. This well-known theory makes it possible to formulate predictions concerning gaming involvement variations from moment to moment; we test these predictions in a relatively simple repeated-measures experiment in which the involvement of the same gamers in the same game at several points in time was measured using a behavioral index supplemented with self-report measures.

### The factors determining involvement in gaming

Playing a game is a process. Even a ‘perfect’ game does not necessarily motivate its users to play it constantly. Instead, it leads to a cycle of gaming sessions separated by other activities. The amount of time spent playing each day varies from survey to survey; however, for example, Hellström and colleagues [16] report that of the more than 7,700 teenagers they surveyed, 6.3% and 10.8% reported playing for over 5 hours on weekdays and at weekends, respectively. This is a significant percentage, but it should be recognized that, apart from individual cases, gaming is not based on continuous multi-hour sessions. It should be assumed that healthy users make cyclical decisions about whether to start or continue playing a game. The question “what factors cause the momentary level of willingness to play?” can be asked at any time of the process. The answer to this question obviously cannot be determined entirely from the static factors described above, otherwise a gamer’s motivation would remain unchanged. Surprisingly, researchers tend to ignore this problem. One of the few exceptions may be the previously mentioned work of Shand [15], who attempted to establish the key motives of people playing online multiplayer video games in three stages: starting, continuing, and ceasing to play. A dynamic view of the motivational processes is important for practical reasons (e.g., keeping users involved in games or avoiding excessive gaming) and for the theoretical perspective (e.g., determining why users abandon games).

According to Malone [26], users are motivated to play games by curiosity and the challenge and fantasy; his explanatory model comprised several components: meaningful goals, sensory curiosity, novel but predictable outcomes, informative feedback and variable difficulty levels. Now, more than forty years after the publication of Malone’s work, the above list may seem obvious. However, we wish to highlight the significance of the two last components: constant feedback that makes the gamer aware that their performance may be the basis of their opinion about their abilities; the varying levels of difficulty are a response to the differences between gamers’ abilities. It is worth noting that Malone in his work intuitively used the concept of a balance between the difficulty of a task and the possibilities of a gamer, but he did not refer to specific theories.

This is why we decided to investigate the specific factors that regulate the desire to play throughout a single gaming session. We are aware of the multitude of factors that potentially influence momentary involvement such as audiovisual stimuli or experiencing the development of a story, but many of them can be difficult to operationalize as a dynamically changing experiences. Our goal was to take the first step by identifying one of these factors. In theory, the perceived relationship between a game’s difficulty and a user’s ability to overcome its challenges should be one of these dynamically changing factors.

### Motivational intensity theory

We believe that Motivational Intensity Theory (MIT) provides one of the most useful theoretical frameworks for attempting to determine the impact of dynamic relative game difficulty levels on a player’s involvement. This theory is being widely used and empirically validated; thus, it provides detailed predictions about the moment of withdrawal of effort put into a task [27, 28]. However, it has not yet been tested in the context of entertainment activities or video games. The main goal of the current paper is to test its applicability to ludic activities.

This theory aims to predict momentary effort mobilization in goal pursuit, therefore it also predicts involvement level [27, 28]. Thus, it is not an alternative to high-level theories focused on identifying motives and goals; instead, it supplements them at the level of the situational factors that determine a player’s involvement at a given moment.

MIT postulates that involvement is primarily controlled by the energy conservation principle, meaning unnecessary effort would be a waste. It uses the term ‘potential motivation’, understood as the maximum amount of effort that is justified for task success. This is defined as being determined by need, incentive value, and outcome expectancy. As a result, potential motivation includes parameters that describe the current situation of the player in the context of stable factors; it can be considered the result of the strength of needs or motives, the value of the goal and the probability of success. Because the cornerstone of this theory is goal pursuit, it may seem that it should not be used to explain gaming behavior as an exclusively intrinsically motivated act. However, previous research has shown that motivation toward gaming is more complex and should be analyzed in terms of needs and motives [3]. So, theoretically, nothing stands in the way of analyzing the dynamics of a single gaming session in MIT terms. The question is whether it is possible to map MIT’s predictions to the context of gaming, and whether these predictions turn out to be right in this context.

This theory formulates different predictions, depending on whether the task requirements are known or not. Both situations may occur in gaming. Since the former seems to be more common in gaming and the MIT predictions are more distinctive as compared to other theories, in this study we decided to focus on situations in which the task requirements are known. In this case, potential motivation serves as the maximum level of momentary involvement. However, the actual involvement is directly determined by the perceived difficulty of the task. Individuals invest as many resources as needed to achieve a goal, but they do not invest more than the potential motivation indicates. To uphold the energy conservation principle, the actor will withdraw from attempts to achieve a goal if the effort necessary to achieve it exceeds their capabilities or the potential benefits; in this case, no effort will be invested.

In a typical experiment testing the predictions of MIT, the participant is instructed to perform a series of tasks with different levels of difficulty (repeated-measures design). The tasks are uninteresting to participants. A question arises: what if the task is enjoyable and motivating? Will the pattern remain unchanged when playing a game? If the answer is ‘yes’, as we expect, this would be the first premise to consider the Motivational Intensity Theory as a useful theoretical framework for research on gamer’s motivation dynamics. This would open a path for further research aimed at objectives such as identification of the optimum levels of difficulty at particular moments of a game or learning curve optimization. In terms of gaming, we expect that if we assume potential motivation to be constant, a gamer’s involvement will increase as the difficulty level increases. Involvement will reach the optimal level when the requirements of the task are close to the potential motivation. After exceeding the level of potential motivation, involvement will drop sharply as a result of an imbalance between motivation and task requirements. We decided to test this MIT-specific prediction in the context of games. We asked participants to play Icy Tower four times with a gradually increasing level of difficulty: from easily achievable by an ordinary student, to impossible to do without numerous attempts. We expected two results. Firstly, we expected a gamer’s involvement to increase in line with the difficulty. Otherwise, we would have to admit that factors other than mere difficulty determine involvement. If the involvement level was stable across the trials, which differ only in terms of difficulty, it would be determined only by the stable characteristics of the game and the user. If it gradually decreased despite the increase in the level of difficulty, this would mean that the effect of the increasing difficulty level was null or not strong enough to overcome the increasing boredom. According to our predictions, involvement will increase from trial to trial until the task becomes too difficult. Secondly, we expected users to withdraw their involvement at the very moment they felt overwhelmed by the task difficulty. Otherwise, we would have to admit that the level of involvement depends on factors other than the level of task difficulty. In other words, it would be a premise for considering that human beings are willing to try to achieve their goals during gaming even if they do not believe that they are possible and worth the effort. In our study we expected to observe a drop in involvement between the 3rd and 4th levels of difficulty, as the 4th level was designed to make the goal unattainable by the average person. Taken together, our predictions were aimed to perform an initial test of MIT’s applicability for further investigations of the dynamics of players’ involvement.

## Materials and method

A written positive opinion of the Research Ethics Committee at the Institute of Applied Psychology of the Jagiellonian University was issued on April 12, 2017.

### Pilot study

A 10-person pilot study was carried out to verify the assumption that controlling the difficulty of the game (Icy Tower, see the description below) by adjusting the pace via the game settings is a sufficient way to manipulate the difficulty level so that it gradually increases to a level that is impossible for non-professional players. Participants were asked to play the game on four difficulty levels (in the game menu, labeled as normal, faster, fastest, insane) for 5 minutes each. Their goal was to reach 100 platforms, and they were also asked to note when they reached 50. They also reported perceived task difficulty and their comments on the difficulty of the game: the lowest level of difficulty turned out to be doable for all participants, while only two participants completed the most difficult level, with one reporting he “got lucky” and did not expect to repeat their result.

### Participants

We formulated two criteria, the fulfillment of which determined the possibility of participating in the study: participants were not allowed to play Icy Tower in the week preceding participation in the study and had to define themselves as non-professional players. From an initial sample of 42 students who voluntarily agreed to participate in the experiment, we excluded 3 because they had played the game we used during the week preceding the study. The final sample contained 39 participants (M_age_ = 23.5, SD_age_ = 6.28, 33 women, 6 men). This research was accepted by the Ethical Committee at the (blinded for review). All subjects gave written informed consent in accordance with the Declaration of Helsinki.

### Procedure

**Fig 1:**
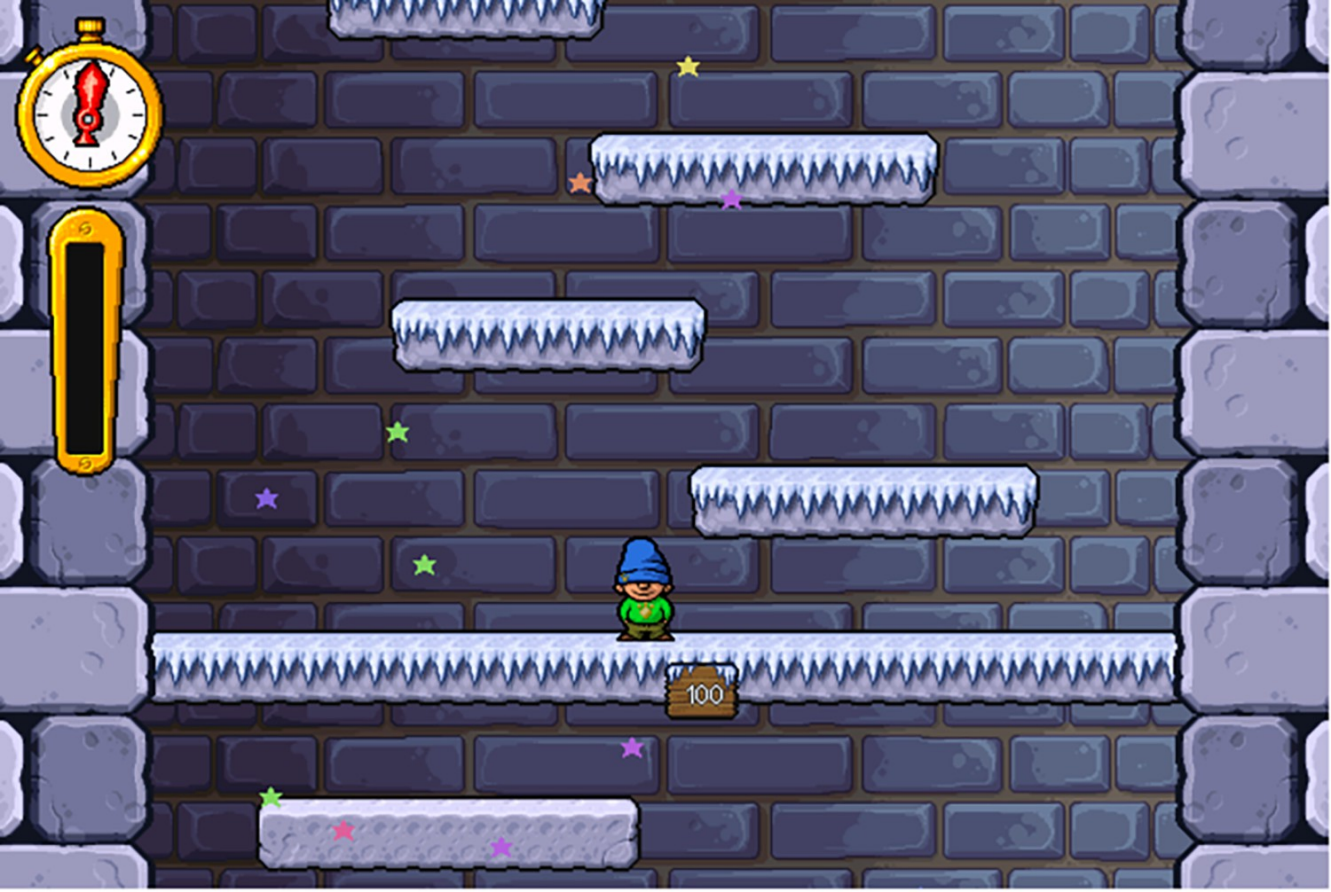
Screenshot from game used in the study (Icy Tower 1.5.1.1). Note: The avatar of the player has just reached the 100th platform (the goal in the study).

The study was conducted in groups of 8 to 14 persons, each of whom were seated at individual workplaces separated by partitions. Each participant in a group began the task at the same time and was not able to observe the actions of others. After completing a form with information about gender, age and experience with games, participants were asked to start a 2-minute training session consisting of free play on the normal difficulty level; after each failed attempt, participants were asked to start over. In the following four experimental sessions, participants were asked to try to reach the 100^th^ platform (easily recognizable, see Fig 1) as many times as possible in 5 minutes. After reaching the goal, participants were supposed to let their character fall down and start another try with the same goal. Subsequent sessions differed in the level of difficulty, which always rose from the lowest to the highest. During each trial, the number of times the spacebar was pressed during the game was counted to evaluate involvement. After each trial, participants were asked to assess the subjective difficulty and their effort on the Overall Workload Scale [29] and the Rating Scale Mental Effort (RSME) [30]. Each participant played four sessions and completed the questionnaires after each trial. Each session was on higher difficulty level than the previous one. When deciding on the fixed order of difficulty levels (previously used, for example, by Roets and colleagues [31]), we were guided by an attempt to maximize the ecological accuracy of the study—in gaming the level of difficulty gradually increases in most of the cases. The difficulty levels were established on the basis of the pilot study. After the session, participants were thanked and invited to contact the research team if needed.

### Materials

#### Icy Tower

As the task, we used the single-player game Icy Tower (version 1.5.1.1), created by the Swedish Free Lunch Design studio. The game ran on Windows-based computers in the university computer lab. This game is a 2D platformer that is controlled with just three buttons, which control horizontal movement and jumping. The goal of the game is to jump up from platform to platform as far as possible in an infinitely tall tower. The platforms constantly move towards the bottom of the screen, thus forcing the player to jump up constantly to avoid falling off the bottom of the screen, which ends the game. After each failure, the player starts again from the bottom. The difficulty level was manipulated via the in-game menu (settings used in study were those labeled Normal, Fast, Faster, Insane).

#### Behavioral involvement index

As a behavioral involvement marker, we used the number of jumps (presses of the space key) per second of active play (after deducting the time required to reload the game after each failure, which varied depending on the number of failures and successes). This is an accurate involvement index in the case of an arcade game that requires the use of only three buttons (space bar and side arrows). It is because of the randomness of platform placement. The only constant requirement is pressing space bar. The number of button presses is directly an investment of resources, because player has to invest cognitive resources (and to smaller extent their body energy) to make decisions about correct moments to press buttons. For this simple game, only one behavior was the right way to increase the pace of the game (i.e., the platforms move downwards faster, therefore the game is more difficult), thus increasing the frequency of spacebar presses. Platforms are positioned very densely so any reduction of jumps could only indicate a decrease in involvement. The amount of spacebar presses is not a direct indication of difficulty level, because it is players’ response to difficulty, which as stated by Motivational intensity theory, isn’t always proportional. It will increase due to increased demand for player’s action, but increasing difficulty even more will result in lowered player’s motivation and their involvement in the game, expressed by decreased number of button presses. The application that was used to record individual button presses was the freeware Keycounter software, created by Jody Holmes. Time spent playing and the number of attempts were recorded using the in-game performance record of each player in each trial.

#### Additional, self-report involvement indices

As additional indices of involvement, we used Polish versions of two popular self-report measures: the Overall Workload Scale [29] and Rating Scale Mental Effort (RSME) [30]. We decided to add the physical effort scale to RSME to emphasize that the object of our interest was mental effort as opposed to physical effort, the latter of which was not meant to be analyzed.

In order to perform a manipulation check, we controlled for how participants perceived the difficulty of the game on a ten-point scale.

## Results

### Manipulation check

**Fig 2:**
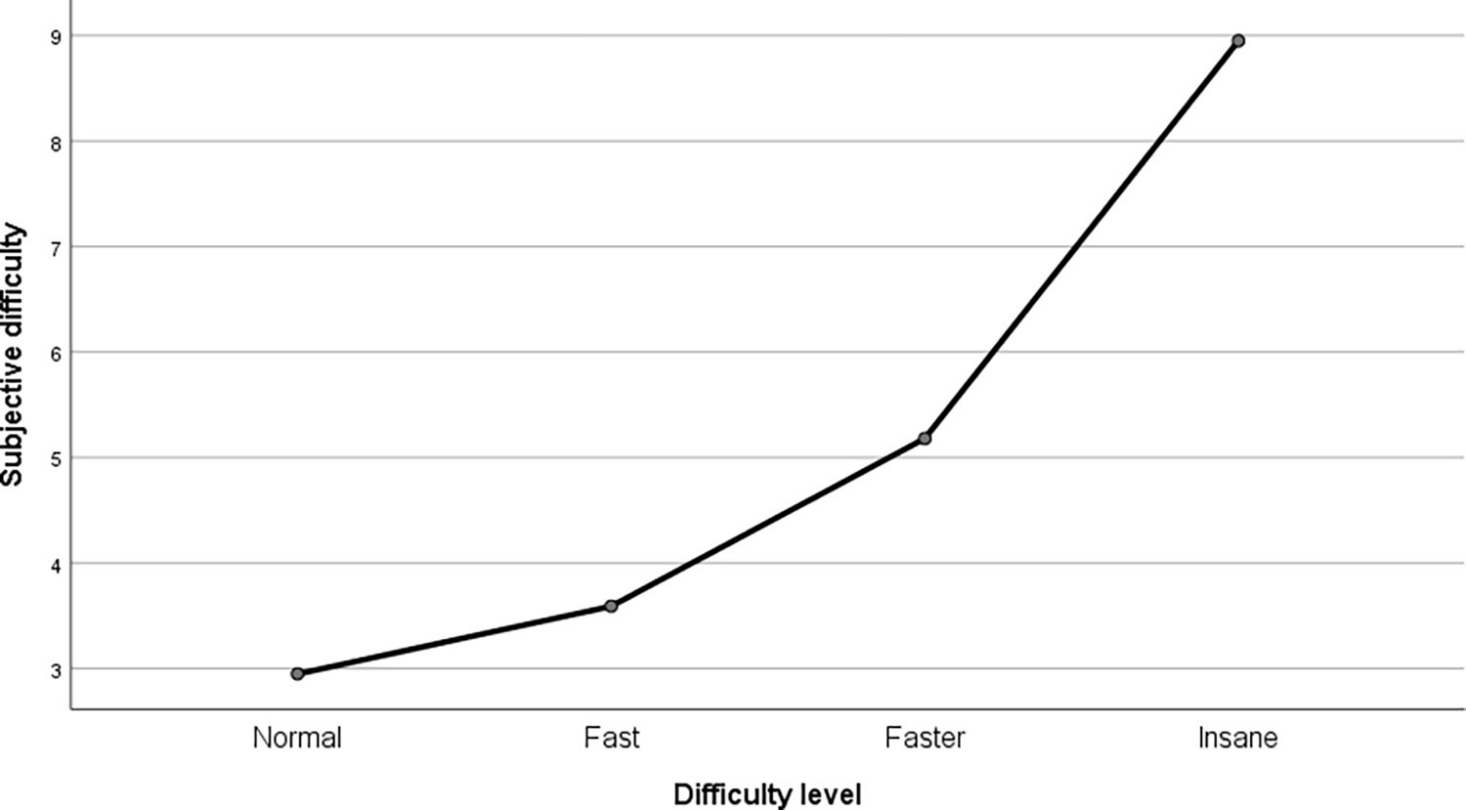
The results of the manipulation check. Subjective difficulty depending on the difficulty level.

We performed a manipulation check in order to compare the difficulty levels of subsequent conditions, which we expected would differ from each other. Due to sphericity assumption violation, we performed repeated-measures analysis of variance (ANOVA) with the Greenhouse-Geisser correction, which showed that the difficulty change was noticed by participants (F (1.94, 73.71) = 152.624, p < .001, partial η^2^ = .801) across all sessions (results are presented in Fig 2). In order to compare subjective difficulty between all the levels, we used Bonferroni correction. Almost all difficulty level differences were statistically significant (except 1st vs 2nd level, whose p-value should be considered as indicating a trend; *p* = .051).

### Subjective involvement across difficulty levels

**Fig 3:**
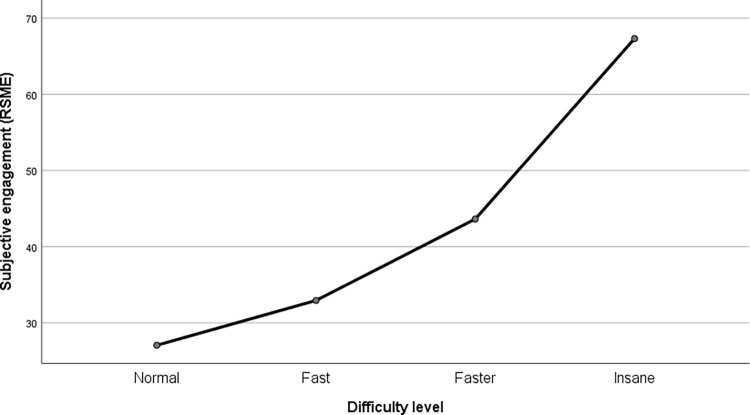
Subjective assessment in the game depending on the level of difficulty–results for Rating Scale Mental Effort.

**Fig 4:**
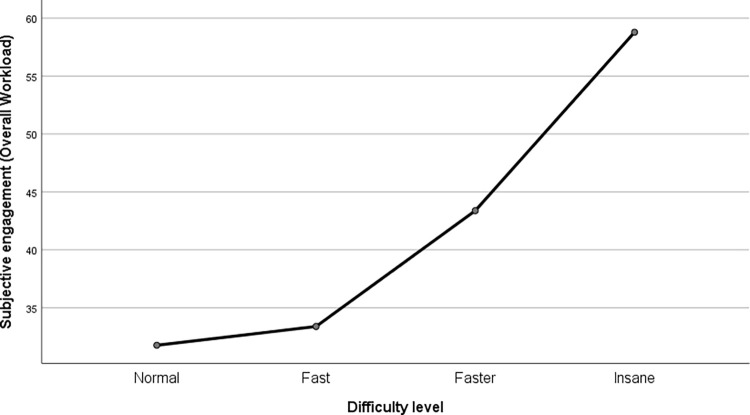
Subjective assessment in the game depending on the level of difficulty–results for the Overall Workload scale.

The repeated-measures ANOVA with Greenhouse-Geisser correction for the Rating Scale Mental Effort showed an increase in declared involvement as the difficulty increased (F (1.801, 61.225) = 36.017, p < .001, partial η^2^ = .514). This trend turned out to be linear (F(1, 34) = 51.003, p < .001, partial η^2^ = .6); the results are presented in Fig 3. For Overall Workload, we found similar results: participants declared increased involvement as the difficulty increased (F (2.022, 72.799) = 24.406, p < .001, partial η^2^ = .404). This trend proved again to be linear (F(1, 36) = 33.52, p < .001, partial η^2^ = .482); the results are presented in Fig 4. The pairwise comparisons with Bonferroni correction confirmed the statistical significance of the differences (except one, between the 1^st^ and 2^nd^ difficulty level) between the means at each difficulty level; the results are presented in Table 1.

**Table 1:** Self-report measures by trial.

Trial	Rating Scale Mental Effort	Overall Workload
1. (Normal)	M = 26.38 SD = 19.713	M = 31.05 SD = 22.76
2. (Fast)	M = 32.51 SD = 20.596	M = 33.08 SD = 19.22
3. (Faster)	M = 42.36 SD = 21.57	M = 42.44 SD = 19.33
4.(Insane)	M = 66.45 SD = 31.23	M = 58.97 SD = 22.86

### Behavioral involvement across difficulty levels

**Fig 5:**
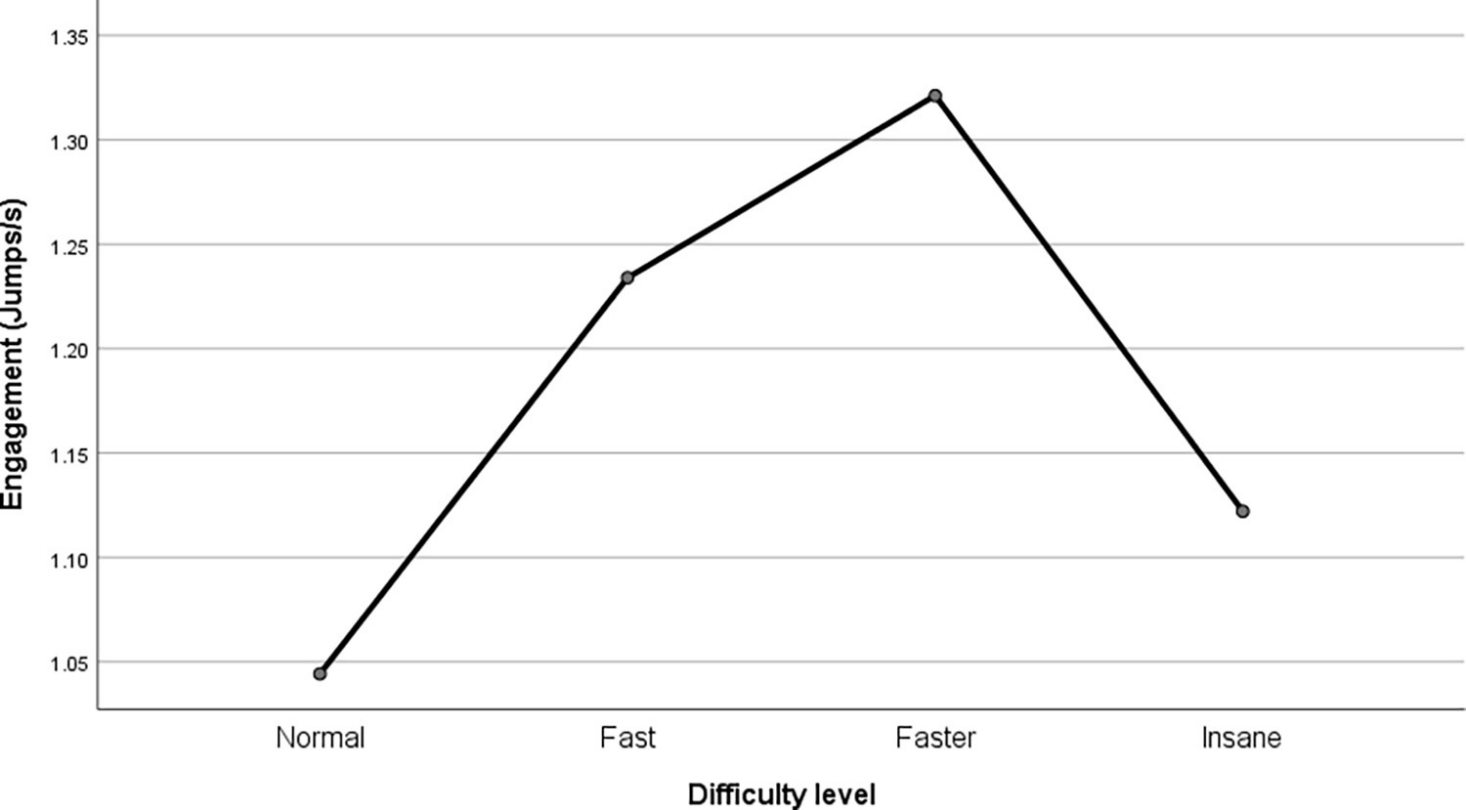
Behavioral index of involvement in the game depending on the level of difficulty–the intensity of space hits (jumps) per second of gameplay.

We conducted the main analysis of the behavioral index of involvement with a repeated-measures ANOVA with Greenhouse-Geisser correction due to violation of the sphericity assumption. The model turned out to be significant (F(2.334, 88.683) = 31.586, p < .001, partial η^2^ = .454). The test of within-subject contrasts showed the expected quadratic trend this time (F(1, 38) = 93.377, p < .001, partial η^2^ = .711); the results are presented in Fig 5. The mean number of jumps per second performed in consecutive trials increased between the three lowest difficulty levels, but after reaching the highest level of difficulty it rapidly decreased to the level of the easiest one. As expected, the pairwise comparisons with Bonferroni correction confirmed the statistical significance of the difference between the means at each level, except for the difference between the easiest and the most difficult level.

## Discussion

The main goal of this paper was to determine whether MIT’s predictions prove themselves in the context of ludic activity, in particular computer games. With use of a fully controlled experiment that is a conceptual replication of the classic experiments conducted in this paradigm, we found that MIT applies not only to unnatural tasks which are generally not interesting (e.g., dynamometer squeezing) but also to natural activities designed to entertain the user. In the case of a game in which the objectives are defined and known to the player, the player’s involvement is determined by the interplay between their potential motivation and task difficulty. This manifests in the phenomenon of involvement increasing as difficulty increases, but only up to the point where the difficulty exceeds the potential motivation. Once this point is reached, a rapid withdrawal of involvement follows. We discuss the details of the results hereunder. MIT predicts that with an increase of difficulty level there will be an increase in involvement, but when it reaches the potential motivation level there will be a sharp decrease in involvement because the motivation is not strong enough to justify expending an increasing amount of energy. This is exactly what we have found: involvement grew from trial to trial until the task became too difficult. Secondly, we expected users to withdraw their involvement at the very moment they felt overwhelmed by the task difficulty. Our results show that the limit is exactly where we expected it to be on the basis of the pilot study, namely between the 3rd and 4th levels of difficulty. Taken together, our results suggest that MIT is a useful framework for further investigations of the dynamics of players’ involvement.

The results we present should be treated as a premise for opening a new direction of research into gamers’ momentary involvement. The fact that a game’s difficulty directly affects the involvement of its users inspires further hypotheses stemming from MIT’s achievements in areas other than games. Even though the assumption that a video game’s difficulty should be adapted to the capabilities of the user seems to be as justified as the assumption that a player’s psychological needs should be met by a game, only the latter has undergone sufficiently detailed research. A study conducted by Juul [32] showed, for example, that players enjoy feeling responsible for their failure, which is a quality that is separate from game difficulty: a game’s “fairness” stems not only from matching the difficulty level to the player’s skill. Considering this, the adequacy of the challenge should not be reduced to self-regulation of the level of difficulty based on a gamer’s decision as this could lead to some of the game’s potential for building involvement being wasted: for example, if a player chooses the wrong difficulty level at the beginning of the game, they may give up playing even though the game could be engaging on the right difficulty level. Considering the results of our study, users may not be able to accurately assess how effortful a game is. As a result, gamers may unknowingly withdraw their effort and then discontinue the game altogether upon seeing an unsatisfactory ratio of achieved results to subjectively assessed (overestimated) commitment. In the context of MIT and our results, systems such as the decades-old gradual increase in the difficulty of the game without player intervention (e.g., *Tetris*) or the relatively new dynamic game difficulty balancing [33] offer a much wider possibility of adjusting the difficulty of a game to potential motivation, thereby engaging the gamer. On the other hand, both these solutions are more demanding for a game developer, therefore further research in the MIT paradigm may help make it easier to create games that take into account the individual needs of the player. Due to individual differences between gamers, a system of gradual increases in the difficulty level may fail to adjust the difficulty curve to individual needs [34]. In this case, not only the results regarding the relationship between difficulty, potential motivation and involvement, but also the broader achievements of the theory, including knowledge about the user’s potential motivation determinants and the possibility of influencing it, may prove helpful [28]. In the case of dynamic game difficulty balancing, there are two basic challenges: which variables should be included in the difficulty-balancing models, and how can these variables be measured? The solutions used so far in the field of models often boil down to the use of fuzzy models, which is sometimes effective [33]. However, taking a step back and looking at the phenomenon from the broader theoretical perspective offered by MIT may in some cases help to see the importance of previously ignored variables, such as fatigue [35], affect [36], success importance [37] and others, whose influence on this complex relationship has been repeatedly proven empirically [38, 39]. MIT may also contribute to the second issue (development of more reliable measurement methods) because detailed relationships between psychological variables (involvement, effort) and their physiological markers have been established during previous research within the framework of this theory. In other words, previous research has already developed a number of methods of measuring involvement in mental tasks on the basis of cardiovascular indicators, including blood pressure, pre-ejection period, heart rate variability and others [38], glucose level in blood [40] as well as behavioral indicators [31]. Thus, providing methods that can be used in the case of gaming involvement research may enrich the possibilities for further research. In light of MIT and our initial results, which prove its applicability, Motivational Intensity Theory could be one of the most useful foundations of future research on gamers’ momentary involvement. It is worth noting that we showed the full pattern of the curvilinear relationship between a game’s difficulty and involvement only for behavioral data. Our participants’ self-reports suggested there is a linear increase in involvement that is unlimited by potential motivation, which contradicts the behavioral data we have collected. This can be explained by imperfections of the self-report methods: it is well known that self-report data on behavioral intentions may be biased due to factors such as self-presentation motives, introspection inability or the limitations of self-report tools [41, 42]. Significantly, the pattern of self-reported involvement is identical to the pattern of difficulty, therefore perhaps the test subjects did not notice the difference between these two concepts. In this light, more information is probably provided by the subject’s actual behavior than by their statements. This may also be another case of guidance for playtesting, as players do not always declare what they actually feel or how they behave.

We would also like to address an issue that could contribute not only to game research but also to MIT research in general: the issue of the dynamics of the decline in involvement and the depth of this decline when the level of potential motivation is exceeded. According to the energy conservation principle, which is the cornerstone of MIT, the decline in involvement should occur rapidly and to a level close to lack of commitment. In our study we showed a significant decrease in involvement; however, due to the discrete nature of the manipulation we cannot clearly state how steep the decrease was; perhaps, if we added an intermediate condition between the 3rd and 4th level, we would see a decrease in involvement in some people after reaching it, while in others this decrease would only occur on the 4^th^ level. Additionally, it is worth paying attention to the fact that this decreased involvement was close to the level of involvement on the easiest level (the difference was statistically insignificant). Because we operationalized involvement as the number of presses of the space bar, it is not clear whether the observed frequency means no involvement at all; in fact, there are presses even in the case of the less engaging trials (the 1^st^ and the 4^th^), which indicates a certain level of involvement. Perhaps this is due to the fact that during the course of the fourth trial the participants gradually realized that it was not possible to achieve the goal, which resulted in a gradual decrease of space hits to an average frequency that was close to that of the 1st level. However, it is also possible that the frequency of space presses remained constant throughout the trial. This result confirms previous studies that questioned the validity of the energy conservation principle, including the meta-analysis of Stanek and Richter [43]. Future studies should pay close attention to these two issues.

Our study also has some limitations which need to be discussed. First of all, one might find it limiting our decision to use the fixed order of difficulty levels, rising from trial to trial. Most MIT studies use a random order of difficulty levels to avoid order effects. However, given that our primary goal was to test if MIT’s assumptions were valid in the gaming context, we had to be careful not to transform playing a game (that was our experimental task) into an "ordinary task". As one of the core features of most games is the gradually increasing difficulty level, we felt we shouldn’t give it up. In this case, we preferred ecological validity to resistance to order effects. However, subsequent studies should also take into account the latter and, balance out the independent factor. The second limitation refers to our decision to model a gaming situation with a well-defined goal. According to MIT, this situation psychologically differs from one in which the user is involved in a game purely on the basis of intrinsic motivation; in this case, we should not expect the goal achievement difficulty to determine the level of involvement because there is no ‘goal’ in the case of an activity that is solely intrinsically motivated. However, even in the context of gaming, a solely intrinsically motivated activity is rare: according to the authors of self-determination theory, “The most basic distinction is between intrinsic motivation, which refers to doing something because it is inherently interesting or enjoyable, and extrinsic motivation, which refers to doing something because it leads to a separable outcome” [44]. Thus, our approach applies to the vast majority of cases in which a player decides to play a game and consciously or unconsciously adopts or formulates the goals he or she wants to achieve. Experimental modelling of other situations in which gaming is solely intrinsically motivated may be challenging, but also these experiments should be conducted in order to establish the potential limitations of MIT. This leads us to another limitation, namely cases in which the goals are not established arbitrarily but by the actor. Having in mind our goal of providing a reason to take MIT into account when considering the dynamics of players’ motivation, we decided to test the most distinctive prediction of this theory, therefore further studies in this paradigm are needed. Thus, we decided to model a situation which has a clear goal. Nonetheless, MIT also provides clear predictions for situations in which activity is intrinsically motivated; in this case, potential motivation instead of task difficulty would be the direct determinant of involvement, but this should be tested in further studies.

Despite these limitations, this study offers the first premise to confirm the sense of using the theoretical framework provided by MIT in gaming research. The participants’ behavior when playing the Icy Tower game confirmed the theory’s predictions regarding the direct relationship between involvement and difficulty level. Involvement increased as the difficulty level of the game increased, but it dropped sharply after the threshold of potential motivation was exceeded. The presented results should contribute to the inclusion of MIT and related methodology into games research. They also represent a significant contribution to the discussion on the fundamental assumption of MIT–the energy conservation principle. In addition, these results should raise game developers’ awareness of how to design involving games and measure involvement. An argument can be made that MIT has already been utilized in games design, albeit without explicit reference to it. An example can be popular game Dark Souls, which while known for its high difficulty, but also allows players to engage in optional cooperation to overcome difficult challenges. With regard to MIT this can be understand as a way of preventing player from reaching the point of frustration when they decide not to invest resources into playing it. What’s more, this use case may suggest further hypotheses resulting from the findings of MIT, which should be tested in the future in the gaming domain. An example of this is the mood-congruency effect, where mood serves as an important cue influencing the global assessment of task difficulty [45]. Outside the domain of gaming, negative mood has been shown to increase the difficulty rating, thereby changing the level of effort. Will there be a similar effect when confronted with a game like Dark Souls? Do constant gaming failures always lower ones mood, which in turn can affect ones effort? With full awareness that the comprehensive verification of all MIT’s hypotheses in the context of games is still ahead of us, we believe that with this report we have taken the first step in this direction.
